# Three cases of anaphylaxis following injection of a depot corticosteroid with evidence of IgE sensitization to macrogols rather than the active steroid

**DOI:** 10.1186/s13601-016-0138-3

**Published:** 2017-01-10

**Authors:** Nicolaj Brandt, Lene H. Garvey, Ulla Bindslev-Jensen, Henrik Fomsgaard Kjaer, Carsten Bindslev-Jensen, Charlotte G. Mortz

**Affiliations:** 1 Department of Dermatology and Allergy Centre, Odense Research Centre for Anaphylaxis (ORCA), Odense University Hospital, University of Southern Denmark, Sdr. Boulevard 29, 5000 Odense C, Denmark; 2 Department of Dermatology and Allergy, Allergy Clinic, Gentofte Hospital, 2900 Hellerup, Denmark

**Keywords:** Macrogols, Anaphylaxis, Corticosteroids, Skin testing, Oral challenge

## Abstract

We present three cases with anaphylaxis after injection of a depot corticosteroid. First, the steroid was suspected as the elicitor, but after evaluation the excipient macrogol was found to be the elicitor. One of the patients had reactions to several unrelated drugs. Increased awareness of anaphylaxis to excipients such as macrogols is needed, especially when allergy tests for the active drug is negative and in patients with a history of repeated anaphylaxis to seemingly unrelated drugs. To establish the correct diagnosis it is important to test with the exact formulation of the culprit drug, as well as all the ingredients including excipients.

## To the editor

Macrogols or polyethylene glycols (PEG) are polymers used as excipients in many different drugs including depot corticosteroid solutions for injection. We present 3 cases of anaphylaxis due to macrogol 3350 after injection of a depot corticosteroid for arthritis or tendinitis. Evaluation, including challenges, with the pure corticosteroid and lidocaine was negative. SPT and specific IgE for latex and chlorhexidine were negative. In all cases skin prick tests (SPT) showed sensitization to macrogol of varying molecular weights, while histamine release (HR) tests to macrogols were negative. Laxatives are the only products on the Danish market declaring the concentration of macrogol 3350. All three patients underwent open titrated oral challenge with the laxative Movicol Junior^R^ containing macrogol 3350 as the active component. Two of the three patients reacted with anaphylaxis. The third patient was only sensitized by SPT to macrogol 6000, and a challenge with a larger dose of macrogol 3350 is planned.

### Case 1

A 46 year-old woman developed plantar pruritus, generalized urticaria, nausea and respiratory symptoms 30 min after injection with Depo-Medrol^R^ (80 mg) and Lidocain^R^ due to Achilles tendinitis. The symptoms progressed with sensation of throat tightness, dysphagia and hypotension and she was treated with intramuscular epinephrine.

One month later, allergy testing was performed: SPT with Depo-Medrol^R^ (containing macrogol 3350) was positive, while SPT with Solu-Medrol^R^ (containing the same steroid component but no macrogol) was negative. Positive SPT to macrogol 6000 and Movicol Junior^R^ (containing macrogol 3350) were found by titrated SPT (Table 1). HR test for both the corticosteroids and macrogols were negative. Titrated intravenous challenge with Solu-Medrol^R^ was negative. Five months after the anaphylactic reaction an open titrated oral challenge with Movicol Junior^R^ was positive with objective signs after the last dose (cumulative dose of 6.56 g macrogol 3350) including itching in the palms, rhinitis and conjunctivitis. Just before the challenge the SPT was repeated with Depo-Medrol^R^ and Movicol Junior^R^. The SPT was still positive for Movicol Junior^R^ but had become negative to Depo-Medrol^R^, and 1 year after the initial anaphylactic reaction both had become negative.

**Table 1:** Diagnostic summary of allergy tests

SPT	Tested substance	Concentration (%)	Case 1	Case 2	Case 3
Corticosteroids	Solu-Medrol^R^	100	neg	neg	neg
	Depo-Medrol^R^*	100	4 mm	neg	neg
	Diprophos^R^*	100	nt	nt	neg
Macrogols	Macrogol 300	100	neg	neg	neg
	Macrogol 400	100	neg	neg	neg
	Macrogol 3000	100	neg	neg	neg
	Macrogol 6000	100	7 mm	neg	5.5 mm
	Macrogol 20,000	0.01	neg	5 mm	nt
	Macrogol 20,000	0.1	neg	9.5 mm	nt
	Macrogol 20,000	1	neg	nt	nt
	Macrogol 20,000	10	neg	nt	nt
Laxative	Movicol junior^R^*	100	5 mm	neg	neg
	Movicol junior^R^*	10	3 mm	neg	nt
	Movicol junior^R^*	1	neg	neg	nt
HR test	Solu-Medrol^R^		neg	neg	nt
	Depo-Medrol^R^*		neg	neg	neg
	Diprophos^R^*		neg	neg	nt
	Macrogol 300		neg	neg	neg
	Macrogol 400		neg	neg	neg
	Macrogol 3000		neg	neg	neg
	Macrogol 6000		neg	neg	neg
	Movicol junior^R^*		neg	neg	neg
Challenge	Lidocain (subcutaneous)		neg	nt	neg
	Movicol junior^R^* (peroral)		pos	pos	neg
	Solu-Medrol^R^ (intravenous)		neg	neg	neg

Underline indicates a positive test

*Pos* positive, *neg* negative, *nt* not tested, *HR* test: Histamin release test

* Contains macrogol 3350, 100% Movicol junior ~104 mg/ml

### Case 2

A 57 year-old man was admitted to the Emergency Department with anaphylactic shock requiring treatment with epinephrine after an intraarticular injection with 80 mg Depo-Medrol^R^ in the knee. Eight years earlier, he developed tachycardia, erythema and general discomfort after a corticosteroid injection in the shoulder with Diprospan^R^ (another steroid drug for injection also containing macrogol 3350) and 4 years earlier he developed respiratory symptoms, palpitations and throat tightness with voice change immediately after a colonoscopy, where he had received pretreatment with Moviprep^R^, an osmotic laxative containing macrogol 3350.

The patient was referred for evaluation 2 years after the reaction to Depo-Medrol^R^. SPT with Depo-Medrol^R^, Diprophos^R^ and Solu-Medrol^R^ were negative and HR test for both the corticosteroids and macrogols were negative (Table 1). A titrated challenge with Solu-Medrol^R^ was negative. SPT was positive for macrogol 20,000 (0.01 and 0.1%) and the patient developed generalized urticaria 2 h after the SPT. He had an open titrated oral challenge with Movicol Junior^R^, and developed a systemic reaction after ingestion of cumulative 916.5 mg of macrogol 3350. He already had subjective symptoms after the first dose, but only developed objective symptoms including urticaria and angioedema immediately after the fourth dose. Six months later, his general practitioner initiated treatment with Escitalopram^R^, containing macrogol 400, and the patient developed urticaria a few minutes after ingestion of the first dose.

### Case 3

A 63 year-old woman with arthritis developed general discomfort, a burning sensation in the soles, erythema spreading to the trunk and arms, respiratory symptoms and mild hypotension after intraarticular injections with 80 mg Depo-Medrol^R^ and Lidocain^R^ in the wrist and finger joints. She was evaluated a few months later. SPT with Depo-Medrol^R^ and Solu-Medrol^R^ were negative, and intravenous challenge with Solu-Medrol^R^ was negative. SPT was positive for macrogol 6000 but negative for other lower molecular weight macrogols including Movicol Junior^R^. HR test for both the corticosteroids and macrogols were negative. The patient had a titrated open challenge with Movicol Junior^R^, which was negative up to 6.56 g of macrogol 3350. A challenge with a higher amount of macrogol 3350 has not been performed due to severe co-morbidity.

## Discussion

During the last year three patients have been referred to our clinic on suspicion of corticosteroid allergy due to anaphylactic reactions after depot corticosteroid injections. All tolerated challenge with the pure corticosteroid, but two were found to be oral challenge positive to the excipient macrogol 3350 while the third was sensitized in SPT. However, in the third case other elicitors were excluded and despite negative challenge with Movicol Junior^R^ up to 6.56 g anaphylaxis to macrogol was still suspected. As the clinical effect of osmotic laxatives is primarily in the gut and <2% is absorbed [1], the challenge dose was probably too low in this patient. Due to comorbidity we have not yet performed challenge with a higher dose of macrogol to verify the allergy. Only few case reports of anaphylaxis to macrogols in corticosteroid solutions can be found in the literature [2–5]. Macrogols are used in many drugs and other products including electrolyte lavage solutions, tablets and topical products, and anaphylaxis have been described to many different formulations via many different administration routes [6]. In patients with severe allergic reactions to chemically unrelated drugs, it is important to consider excipients such as macrogols as potential culprits. Unfortunately, this is rarely considered, and patients may have several severe reactions before macrogols (or other excipients) are suspected as in case 2. When anaphylaxis to the active drug is excluded in patients with reactions to unrelated drugs, there is a risk of labelling these cases “idiopathic anaphylaxis”, if the excipients in the drugs administered are not considered. In such cases the patient is at risk of future anaphylaxis on re-exposure to the excipients.

The sensitivity and specificity of skin tests and HR test evaluated in relation to the outcome of oral challenge to macrogol are unknown. As systemic reactions to skin testing with macrogols have been reported in several cases [6], it is important to titrate the skin test especially when including the high molecular weight macrogols, and furthermore, to extend observation time to 30 min as development of the wheal may be delayed [6]. Case 2 reacted to only high molecular weight macrogol in SPT, but was challenge positive to macrogol 3350. This finding supports the hypothesis, that clinical reactivity to macrogols is related to both amount and molecular weight, and although sensitization may occur to lower molecular weight macrogols, which more readily penetrate skin and mucosa, high molecular weight macrogols may subsequently elicit a response at lower concentrations [6]. This has implications for the skin test concentrations used, and it may be that SPT with higher molecular weight macrogols can be used to diagnose cases with reactions to lower molecular weight macrogols in the more distant past, as seemed to be the case in case 2. Another important finding from these cases is that skin reactivity may decrease or even be lost over time as has been shown regarding specific IgE for other allergens such as penicillin and chlorhexidine [7, 8]. Case 1 was skin tested 3 times during the first year, first the SPT was positive and then SPT reactivity diminished over time although challenge remained positive. Case 2 was not evaluated until 2 years after the last anaphylactic episode, and SPTs besides macrogol 20,000 were negative at this time, but challenge with macrogol 3350 (Movicol Junior^R^) was positive. HR test was negative in all cases, and was not helpful to predict a reaction to macrogol in our cases. The same has been shown in other studies for both HR test and basophil activation test in relation to macrogol allergy [6].

## Abbreviations

PEG: polyethylene glycols
SPT: skin prick tests
HR: histamine release
